# Therapeutic Potential of Cannabinoids in Glaucoma

**DOI:** 10.3390/ph16081149

**Published:** 2023-08-14

**Authors:** Theresa Lindner, Doreen Schmidl, Laura Peschorn, Viktoria Pai, Alina Popa-Cherecheanu, Jacqueline Chua, Leopold Schmetterer, Gerhard Garhöfer

**Affiliations:** 1 Department of Clinical Pharmacology, Medical University Vienna, 1090 Vienna, Austria; theresa.lindner@meduniwien.ac.at (T.L.); doreen.schmidl@meduniwien.ac.at (D.S.); laurapeschorn@rsu.edu.lv (L.P.); viktoria.pai@meduniwien.ac.at (V.P.); leopold.schmetterer@meduniwien.ac.at (L.S.); 2 Department of Ophthalmology, Emergency University Hospital, 050098 Bucharest, Romania; alina_cherecheanu@yahoo.com; 3 Carol Davila University of Medicine and Pharmacy, 020021 Bucharest, Romania; 4 Singapore Eye Research Institute, Singapore National Eye Centre, Singapore 169856, Singapore; jchua@duke-nus.edu.sg; 5 Ophthalmology and Visual Sciences Academic Clinical Program, Duke-NUS Medical School, Singapore 169857, Singapore; 6 SERI-NTU Advanced Ocular Engineering (STANCE), Nanyang Technological University, Singapore 639798, Singapore; 7 School of Chemistry, Chemical Engineering and Biotechnology, Nanyang Technological University, Singapore 637459, Singapore; 8 Center for Medical Physics and Biomedical Engineering, Medical University Vienna, 1090 Vienna, Austria; 9 Institute of Molecular and Clinical Ophthalmology, 4031 Basel, Switzerland

**Keywords:** cannabinoids, cannabinoid receptors, endocannabinoid system, glaucoma, intraocular pressure, neuroprotection, ocular hemodynamics, ocular blood flow

## Abstract

Glaucoma is a leading cause of irreversible blindness worldwide. To date, intraocular pressure (IOP) is the only modifiable risk factor in glaucoma treatment, but even in treated patients, the disease can progress. Cannabinoids, which have been known to lower IOP since the 1970s, have been shown to have beneficial effects in glaucoma patients beyond their IOP-lowering properties. In addition to the classical cannabinoid receptors CB_1_ and CB_2_, knowledge of non-classical cannabinoid receptors and the endocannabinoid system has increased in recent years. In particular, the CB_2_ receptor has been shown to mediate anti-inflammatory, anti-apoptotic, and neuroprotective properties, which may represent a promising therapeutic target for neuroprotection in glaucoma patients. Due to their vasodilatory effects, cannabinoids improve blood flow to the optic nerve head, which may suggest a vasoprotective potential and counteract the altered blood flow observed in glaucoma patients. The aim of this review was to assess the available evidence on the effects and therapeutic potential of cannabinoids in glaucoma patients. The pharmacological mechanisms underlying the effects of cannabinoids on IOP, neuroprotection, and ocular hemodynamics have been discussed.

## 1. Introduction

Cannabis or marijuana is one of the most widely used drugs in the world. In many countries, cannabis remains an illegal drug, while other countries allow medical use or consumption. Currently, there is a growing interest in cannabis and both medical and recreational use is gradually being legalized [1]. The cannabis plant, *Cannabis sativa*, has been used as a medicinal and psychotropic drug for many centuries [1,2,3]. It is one of the earliest cultivated plants, domesticated around 12,000 years ago [1,2]. It was first cultivated in Central or Southeast Asia [2]. Cannabis provided fibers for ropes and nets, food, and seeds for oil [1,3]. Early civilizations, including China, Egypt, Greek, India, and the Roman Empire also used Cannabis for its medicinal and psychoactive properties [1]. In the twentieth century, the psychoactive effects of tetrahydrocannabinol (THC) led to the prohibition of cannabis use in the United Kingdom with the “Misuse of Drugs Act 1971” and in the United States with “The Marihuana Tax Act” in 1937, as the drug was considered to be significantly harmful and had no medicinal use. [2,3]. To date, cannabinoids exhibit therapeutic potential for chronic pain, spastic disorders, such as those associated with multiple sclerosis, unwanted weight loss, and nausea and vomiting resulting from chemotherapy [4,5]. The potential use of cannabinoids in glaucoma due to their hypotensive effect on intraocular pressure (IOP) has been explored since the 1970s [6,7]. Several studies have investigated the effects of cannabinoids in glaucoma in terms of their IOP-lowering and potential neuroprotective effects [8,9,10,11,12]. As public and scientific interest of cannabinoids has increased in recent years, there is a growing need for a better understanding of the ocular mechanisms underlying cannabinoids. Therefore, we have provided an overview of the current evidence on the effects and therapeutic potential of cannabinoids in glaucoma.

## 2. Glaucoma

Glaucoma is the leading cause of irreversible blindness worldwide, affecting approximately 70 million people, with its prevalence predicted to further increase in the future [13]. Glaucoma is an umbrella term for multifactorial optic nerval neuropathies associated with the progressive loss of retinal ganglion cells (RGCs) resulting in characteristic visual field defects [14]. Despite the burden on affected patients, glaucoma also imposes high economic costs, especially in the late stages [15]. Therefore, early diagnosis and adequate treatment are vital to prevent future disability. An elevated IOP is considered to be the most important risk factor underlying the development and progression of the disease and lowering the IOP remains the only proven treatment option [16]. Drug therapies, such as prostaglandin derivatives, β-receptor antagonists, α2-receptor agonists, carbonic anhydrase inhibitors, and cholinergic agonists, are used to lower IOP as a single or combined application [17,18,19]. In 2017, the US Food and Drug Administration (FDA), and more recently the European Medical Agency (EMA), approved the Rho-kinase inhibitor netarsudil to lower elevated IOP in patients with open-angle glaucoma or ocular hypertension [18,19]. Rho-kinase inhibitors are a new class of ocular hypotensive drugs that exhibit cytoskeletal modulatory effects, inducing their effect mainly through increased aqueous humor outflow via the trabecular meshwork and adjacent tissues [19].

Although several anti-glaucoma medications and surgical procedures are currently available to lower IOP, a considerable number of patients progress despite adequate treatment [20]. Therefore, therapy aimed at more than just lowering IOP would be required [17]. However, despite the identification of IOP as the main risk factor for progression, the molecular mechanisms for optic nerve damage in glaucoma are not fully understood. Since a significant number of primary open-angle glaucoma patients do not have a statistically elevated IOP, this suggests the contribution of other mechanisms than IOP to optical neuropathy [21]. Among others, potential contributing mechanisms involve tissue ischemia caused by vascular dysregulation, inflammatory mediators, including oxidative stress, and abnormal effects of endogenous substances, such as glucocorticoids, glutamate, nitric oxide, endothelin, neurotrophic factors, and caspases [22,23,24]. These pathomechanisms have not yet been understood in detail, but therapies targeting components other than IOP are actively being researched. Currently, nutrients and nutraceuticals with anti-apoptotic, anti-inflammatory, and anti-oxidant properties, like vitamins, palmitoylethanolamide (PEA), melatonin, citicoline, coenzyme Q10, or ginkgo biloba extract, are being investigated for their neuroprotective effect in glaucoma therapy [25,26]. Available data from previous studies have shown that nutritional supplementation may play a role as a coadjutant in the therapeutic management of glaucoma, but the clinical significance of these data remains to be demonstrated [25]. As cannabinoids are thought to have effects beyond lowering IOP, this review evaluates their potential role in glaucoma treatment.

## 3. Marijuana and Endocannabinoids

**Figure 1:**
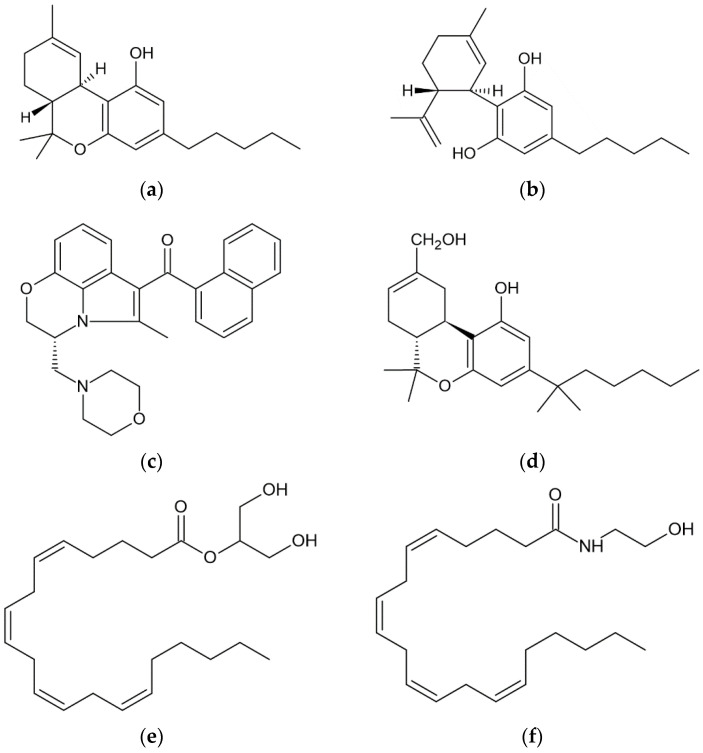
Chemical structures of six cannabinoids: the phytocannabinoids (**a**) tetrahydrocannabinol (THC) and (**b**) cannabidiol (CBD); the synthetic cannabinoids (**c**) WIN55212-2 and (**d**) HU-210; and the endocannabinoids (**e**) 2-arachidonylgylcerol (2-AG) and (**f**) arachidonoyl ethanolamide (anandamide, AEA).

Cannabinoids can be classified into phytocannabinoids, synthetic cannabinoids, and endocannabinoids (eCBs, Figure 1). The term “cannabinoids” is used for any chemical substance that binds to cannabinoid receptors and has effects similar to those produced by the cannabis plant [27].

### 3.1. Phytocannabinoids

*Cannabis sativa* L. (*C. sativa*), contains about 120 phytocannabinoids, of which 11 different chemical classes have been identified [28]. Phytocannabinoids are mainly found in the resin secreted by the trichomes of female plants [3]. The most studied ones are Δ^9^-tetrahydrocannabinol (Δ^9^-THC) and cannabidiol (CBD) [29]. They bind to cannabinoid receptors resulting in their characteristic clinical or psychoactive effects [29]. Their targets include the classical cannabinoid receptors type 1 and 2 (CB_1_ and CB_2_), along with numerous other targets [29].

From a pharmacological perspective, Δ^9^-THC acts as partial agonist at the CB_1_ and CB_2_ receptors [28,29]. As characteristic of a partial agonist, Δ^9^-THC exhibits agonist–antagonist effects. Whether Δ^9^-THC exerts more agonistic or antagonistic effects depends on the proportion of cannabinoid receptors in the tissues, the presence of other cannabinoid receptor agonists, and the species [28]. Δ^9^-THC exhibits neuroprotective, anti-spasmodic, and anti-inflammatory effects [30]. The psychotropic effects of cannabinoids and the cannabinoid-induced “tetrad” that includes hypolocomotion, hypothermia, catalepsy, and analgesia are attributed to the partial agonist activity of Δ^9^-THC at CB_1_ receptors [28,29,30,31].

Since the psychotropic effects of Δ^9^-THC are of concern, there is growing interest in cannabinoids without psychotropic effects, like CBD or cannabidivarin (CBDV). Anti-inflammatory, anti-oxidative, anti-convulsive, analgesic, anti-emetic, and neuroprotective effects have been described for CBD [30]. Other pre-clinical studies showed anti-tumor, anti-invasive, and anti-angiogenic effects in cancer research [30]. CBD is known to have a low affinity for CB_1_ and CB_2_ receptors and to act as an inverse agonist of the classical cannabinoid receptors [28,30]. There is evidence that CBD acts as a negative allosteric modulator of CB_1_ at concentrations lower than those required to act as an inverse agonist. In this way, CBD does not activate the receptor itself but reduces the potency and efficacy of orthosteric ligands, such as Δ^9^-THC [32]. CBD has also been shown to act as a negative allosteric modulator of CB_2_ receptors [33]. These results implicate that CBD could be used to counteract several adverse effects of Δ^9^-THC and increase the therapeutic index of Δ^9^-THC [29].

### 3.2. Synthetic Cannabinoids

Synthetic cannabinoids are a structurally diverse group of psychoactive substances produced by chemical means [27,34]. In recent years, more than 450 synthetic compounds have been synthesized and often these substances bear the initials of the person/institution that performed their synthesis [27]. They can be chemically classified into classical, non-classical, aminoalkylindoles, eicosanoids, hybrids, and others [34]. Synthetic cannabinoids from the eicosanoid class are structurally similar to eCBs, whereas classical synthetic cannabinoids are more similar to phytocannabinoids [34]. This group of substances can bind to both cannabinoid and non-cannabinoid receptors, but has a higher affinity for cannabinoid receptors than Δ^9^-THC as they are mostly full agonists [34]. This results in greater potency, but also stronger psychoactive effects [27,34]. The FDA has approved three synthetic cannabis-related drug products which contain dronabinol and nabilone, as well as one cannabis-derived drug product containing CBD [35]. Cannabis-related compounds are created in a laboratory setting and some synthetic compounds may also occur naturally in the plant, such as dronabinol, while others are exclusively synthetically produced,, such as nabilone [35]. In contrast to cannabis-related compounds, cannabis-derived compounds, such as CBD and THC, are extracted directly from the cannabis plant [35]. The product containing CBD has been approved for the treatment of seizures associated with Lennox–Gastaut and Dravet syndrome [35]. Dronabinol and nabilone are used to treat nausea associated with cancer chemotherapy and/or anorexia associated with weight loss in AIDS patients [35]. While not a synthetic cannabinoid, a cannabis extract oral spray, containing equal quantities of THC and CBD, has been approved in Canada, New Zealand, and several European countries for the treatment of spasticity associated with multiple sclerosis when the patients have an inadequate response to other treatments or cannot tolerate their side effects [36].

### 3.3. Endocannabinoids

The endocannabinoid system (ECS) in the eye refers to a complex network of signaling molecules, receptors, and enzymes that play a crucial role in maintaining ocular homeostasis and in regulating various physiological processes [37]. Within the eye, the ECS is found in different ocular structures, including the cornea, conjunctiva, retina, and various cells, such as the RGCs, ciliary body, and trabecular meshwork [37,38,39,40]. Activation of the ECS in these structures can modulate various functions, including inflammation, IOP, neurotransmission, photoreception, and vascular regulation. Considering the pharmacological aspect, the ECS comprises endogenous cannabinoids (endocannabinoids, eCBs), cannabinoid receptors, and the proteins that transport, synthesize, and degrade eCBs [41,42]. In recent years, it has become clear that the ECS is more complex than originally thought as the number of identified components has increased. The most studied eCBs are arachidonoyl ethanolamide (anandamide, AEA), 2-arachidonoylglycerol (2-AG), and PEA, an anandamide congener [8,37,40]. As stated above, eCBs are present in many parts of the mammalian eye, containing the retina, choroid, iris, ciliary body, and trabecular meshwork [40]. In glaucoma patients, there is evidence that concentrations of 2-AG and PEA are decreased in the ciliary body, suggesting that eCBs may involve IOP regulation [43]. While 2-AG reacts as a full agonist and AEA as a partial agonist for the CB_1_ and CB_2_ receptors [44], PEA only binds them with weak activity [45]. eCBs and eCB-like molecules target the receptors GPR18, GPR55, GPR119, TRPV1, and PPARα-γ with different binding activity [45]. In order to maintain the balance of eCBs in the body, they are synthesized or degraded as needed [40]. eCBs are synthesized from membrane phospholipids by diacylglycerol lipase (DGL) α/β and N-arachidonoyl phosphatidylethanolamine phospholipase D (NAPE-PLD) [30]. Degradative enzymes mainly include fatty acid amide hydrolase (FAAH) and monoacylglycerol lipase (MAGL), but also cyclooxygenase-2 (COX-2) and lipoxygenases (LOXs) [30]. Synthesizing and degrading enzymes have been described in the retina and trabecular meshwork [40]. This indicates IOP modulating and neuroprotective capacities via the activity of metabolizing enzymes, such as COX-2, FAAH, and MAGL [37,46]. The targets of eCBs and phytocannabinoids overlap to some extent [42]. An overview to the interactions of phytocannabinoids with the ECS system has been outlined in the excellent review of Di Marzo and Piscitelli [42].

## 4. Molecular Targets and Mechanisms for Cannabinoid-Induced Ocular Effects

So far, two main types of cannabinoid receptors, CB_1_ and CB_2_, have been identified as promising therapeutic targets. In addition to binding to the classical CB_1_ and CB_2_ receptors, cannabinoids and their metabolites also interact with non-cannabinoid receptors. The following overview presents the most important cannabinoid and non-cannabinoid receptors with a specific focus on glaucomatous disease.

### 4.1. CB_1_ and CB_2_ Receptors

CB_1_ and CB_2_ receptors are the natural receptors for eCBs and can interact with phytocannabinoids and synthetic cannabinoids. The CB_1_ receptor was first discovered back in 1990 [47]; a second CB_2_ receptor was successfully cloned in 1993 [48]. Pharmacologically, the CB_1_ and CB_2_ receptors are members of the G protein-coupled receptor family. They are defined by seven transmembrane-spanning helices with intervening intracellular loops and a C-terminal domain that can couple with G proteins. Signal transduction is mostly negatively stimulated via adenylyl cyclase and positively stimulated to mitogen-activated protein kinase (MAPK) [49,50]. CB_1_ further regulates ion channels, such as potassium and calcium channels [49,50]. CB_1_ receptors are mainly found in the human central nervous system (CNS) and are involved in the release of neurotransmitters [29,51]. The CB_1_ receptor is also known to be the main receptor underlying psychotropic effects [52]. CB_2_ receptors are predominantly present in peripheral tissues, such as the immune system, and regulate the release of cytokines [29,53].

In the human eye, CB_1_ receptors have been detected in the cornea, iris, ciliary body including the epithelium, ciliary muscle and blood vessels of the ciliary body, trabecular meshwork, Schlemm’s canal, and retina [54]. The location and critical anatomical distribution of cannabinoid receptors indicate that cannabinoids may influence IOP through both increasing aqueous humor efflux and decreasing aqueous humor production [55]. A possible influence of cannabinoids on conventional aqueous humor outflow has been suggested by the existence of CB_1_ receptors in the trabecular meshwork and in the canal of Schlemm [56]. Aqueous humor production and/or uveoscleral outflow imply an effect via the CB_1_ receptors in the ciliary pigment epithelium and ciliary muscle [56]. The distribution and precise roles of CB_2_ receptors in the human eye appear to be less well known, but CB_2_ receptors have been found in the cornea, trabecular meshwork, and retina, particularly in animal models [57,58,59,60].

In the retina, CB_1_ receptors have been detected in the ganglion layer, the inner and outer plexiform layers, the inner nuclear layer, and the outer segments of photoreceptors [54,60]. CB_2_ receptors have also been described in retinal cell types and layers, like amacrine, bipolar, Müller, microglial, and RGCs, or in the retinal pigment epithelium [59,60]. CB_1_ and CB_2_ receptors are responsible for the functional modulation of neuroretinal cells in animal tissues and may therefore be a potential target for neuroprotection [60].

### 4.2. Non-Cannabinoid Receptors

The most commonly described G protein-coupled receptors, in addition to the CB_1_ and CB_2_ receptors, to which cannabinoids bind are GPR18, GPR55, and GPR119. GPR55, also known as the putative “type 3” cannabinoid receptor, interacts with eCBs, phytocannabiniods, and synthetic cannabinoids [61,62]. CBD binds GPR55 and GPR18 as an antagonist, while Δ^9^-THC acts as an agonist [5]. GPR55 has been described to be present in the trabecular meshwork and rods of the retina [58,63,64]. Its signaling pathway differs from the classical cannabinoid receptors by activating Ras homolog gene family member A (Rho A) and Rho-associated protein kinase (ROCK) [62]. GPR55 is widely distributed in many tissues, including in the CNS and immune system. Showing anti-inflammatory properties, GPR55 and CB_2_ receptors can cross-talk to regulate neutrophil responses [65]. GPR18, a G protein-coupled receptor, can be activated by N-arachidonoyl glycine, a carboxylic metabolite of the endocannabinoid AEA, or phytocannabinoids, such as Δ^9^-THC or CBD [66,67]. GPR18 has been found to be expressed in the ciliary and corneal epithelium, the trabecular meshwork, and retina [68,69]. In addition, GPR18 mRNA and protein were found in the endothelium of retinal vessels, suggesting its contribution to cannabinoid-mediated retinal vasoactivity [69]. GPR119, an orphan receptor for endogenous lipids with eCB-like structures, has not been described in detail for the eye, as its interest is more in the regulation of insulin secretion. Protein expression of GPR119 is most pronounced in structures near the chamber angle associated with sites of outflow, including the trabecular meshwork, and GPR119 has also been shown to modulate IOP in female mice [70].

Cannabinoids interact with transient receptor potential (TRP) ion channels. The TRP protein superfamily are trans-membrane proteins which are mostly expressed in the nervous system and are involved in cellular processes related to mechano-, chemo-, thermo-, and photosensation, and ion homeostasis [71,72]. TRP channels mainly control the flux of calcium across the plasma membrane and influence other ion channels sensitive to calcium [72]. Currently six TRP channels can mediate cannabinoid activity, including TRPV1–4 (TRP vanilloid), TRPA1 (TRP ankyrin), and TRPM8 (TRP melastatin) [72]. Δ^9^-THC acts with TRPV2–4 channels as an agonist, while CBD also binds to TRPV1 and TRPV2 as an agonist, indicating that the affinity for TRP receptors depends on the selected cannabinoid [5,73]. TRPV1, a capsaicin-sensitive vanilloid TRP subunit 1, can be targeted by the endocannabinoid AEA and is expressed in the retina, including the RGCs and retinal microglia [74]. TRPV4 has been reported as a promising molecular target of glaucoma as it is expressed in the trabecular meshwork showing a crucial role in IOP regulation [75,76], and is also found in RGCs and retinal plexiform layers [77,78]. TRPV1 and TRPV4 have been found in the optic nerve head, indicating that calcium handling may influence optic nerve function [79]; their activation leads to the apoptosis of RGCs [74,77,78,80].

It has been suggested that not only the classical cannabinoid receptors regulate anti-inflammatory effects. Nuclear peroxisome proliferator-activated receptors (PPARs) are members of the nuclear receptor superfamily. PPARs are ligand-regulated transcription factors that control gene expression related to inflammation, glucose and lipid metabolism, metabolic homeostasis, adipogenesis, and cell proliferation [81,82]. Both Δ^9^-THC and CBD exhibit agonistic effects on PPARγ [5]. In addition to the aforementioned receptors, CBD can decrease pain scores and neutrophil infiltration in corneal hyperalgesia through the involvement of serotonin (5-HT) receptors and reduce the release of TNF-α in retinal microglia via adenosine receptors [83,84,85]. The interaction of cannabinoids and their metabolites with numerous receptors makes understanding their mechanisms of action complex and the development of new therapies difficult.

## 5. Therapeutic Approaches of Cannabinoids on IOP

As IOP is the only modifiable risk factor that can slow down or prevent the progression of the disease, pharmacological IOP-lowering is still the mainstay of glaucoma therapy. Pharmacological targets of IOP-lowering drugs include aqueous humor production and aqueous humor outflow facility via the trabecular meshwork as well as the uveoscleral pathway [17].

There is evidence that cannabinoids decrease aqueous humor production and increase trabecular and uveoscleral outflow, thereby affecting IOP [86]. As previously described, IOP-lowering effects appear to be mainly mediated via the CB_1_ receptor [8,56]. In contrast to the CB_1_ receptor, the CB_2_ receptor does not appear to mediate IOP-lowering properties [87]. Other mechanisms that contribute to lowering the IOP are the contraction of the ciliary muscle and reduced secretion from the ciliary process through decreasing the release of noradrenaline [55]. Beyond this, the metabolites of the ECS are reported to lower the IOP due to the induction of COX-2-dependent prostaglandins and matrix metalloproteinases in the non-pigmentary ciliary epithelium [46]. It has been demonstrated that AEA and Δ^9^-THC can act as COX-2-inducing cannabinoids [46]. Via the cyclooxygenase (COX) pathway, the hydrolysis of eCBs to arachidonic acid, the precursor of prostanoid synthesis, occurs [56]. COX-2 oxidates AEA and 2-AG into a series of prostaglandin ethanolamides (prostamides) and prostaglandin glyceryl esters [88]. These are similar to the prostaglandins formed from arachidonic acid [88]. Prostamides exert potent effects in glaucoma, such as the prostamide analogue bimatoprost [88]. Prostamides and prostaglandins exert effects through the uveoscleral signaling pathway [37,89]. COX-1 and COX-2 are normally found in the non-pigmented secretory epithelium of the ciliary body [90]. However, a specific loss of COX-2 occurs in patients with primary open-angle glaucoma [90]. Cannabinoids stimulate the expression of COX-2 and matrix metalloproteinases [46]. These mechanisms induce improved outflow using increased dimensions of Schlemm’s canal or through remodeling the extracellular matrix [46,86,91,92].

### 5.1. Clinical Studies on the IOP-Lowering Effect of Cannabinoids

There have been several human studies on healthy subjects and patients with ocular hypertension and glaucoma which executed the effect of cannabinoids, especially Δ^9^-THC, on IOP [8,10]. These studies indicated that not all cannabinoids show the same effect on IOP [8,10]. The reason for this differing effect has not yet been fully elucidated but may be at least partially related to the complex interaction between IOP regulation and cannabinoid receptors [67]. Different routes of administration have been used, including oral, inhalation, intravenous intake, and topical application [8,10]. One of the first studies assessing the IOP-lowering potential of cannabinoids was conducted in 1971 by Hepler and Frank. The authors of this study reported an approximately 25% decrease in IOP in 11 healthy subjects one hour after smoking 18 mg of Δ^9^-THC [6]. This IOP-lowering capacity of systemically administered THC has later been confirmed in both glaucoma patients and in healthy subjects [8]. In the following, we provide a brief overview on the literature concerning the effects of cannabinoids on IOP. A more detailed overview of the IOP-lowering potential of cannabinoids has been provided in the previously published comprehensive reviews of Passani et al. [8] and Wang and Danesh-Meyer [10].

Most clinical trials have examined orally administered THC. The advantage of orally administered cannabinoids is that exact dosing of the drug is possible. Bioavailability shows 10–20% as there is a high first-pass metabolism [10,88]. Δ^9^-THC has been administered in doses ranging from 5 mg to 80 mg in previous studies [8]. As such, a variety of clinical studies have investigated the effects of oral Δ^9^-THC and synthetic cannabinoids, such as nabilone, BW146Y, or dronabinol, and demonstrated transient ocular hypotensive effects [93,94,95,96,97]. This IOP-lowering effect was at its strongest 2–4 h after administration, reaching approximately 10–30% [8]. This absolute change in IOP seemed to be a dose–response relationship showing greater IOP reductions from baseline with increased dosage, but no dose–duration relationship [98,99]. However, these studies only reported transient effects of cannabinoids, raising the issue that frequent daily doses are required for a therapeutic effect. Based on the IOP-lowering effects of 3–4 h [95], medical marijuana would need to be consumed at least 6–8 times a day and glaucoma patients would be placed at risk for substance dependence [99].

In contrast to oral Δ^9^-THC, orally administered CBD failed to lower IOP. In a randomized, double-masked, placebo-controlled four-way crossover study by Tomida et al., patients with ocular hypertension or early primary open-angle glaucoma received a single sublingual dose of 5 mg Δ^9^-THC, 20 mg CBD, 40 mg CBD, or placebo [95]. In this study, CBD did not exert an IOP-lowering effect, and the higher dose of 40 mg CBD even resulted in a transient increase in IOP 4 h after administration [95]. However, the latter study was limited to only six subjects [95]. Further evidence by a study from Miller et al. indicated that Δ^9^-THC lowered IOP in mice, whereas CBD had opposite effects and interfered with the Δ^9^-THC effects [67]. This can be attributed to the effect of CBD acting as a negative allosteric modulator at CB_1_ receptors and should be considered as a potential side effect in concern of long-term use [67]. A more recent interest has also developed towards orally administered PEA; it showed IOP-lowering effects in patients with ocular hypertension, glaucoma, and after prophylactic iridotomy [100,101,102].

In addition to orally administered cannabinoids, intravenous and inhaled applications of Δ^9^-THC showed ocular hypotensive effects [8]. Dosages of inhaled Δ^9^-THC ranged from 12 mg to 80 mg and IOP reduction ranged from 13% to 34%, respectively [8]. The maximum IOP-lowering effect was reached approximately 90 min after inhaled administration [8]. Inhalation of cannabinoids is limited by their variable bioavailability reaching from 2% to 56% depending on the frequency of puffs, depth of inhalation, and breath hold [10,103]. This makes inhalation difficult for clinical use. Intravenous administration has only been tested on 12 healthy subjects and not yet on glaucoma patients. At doses of 0.022 mg/kg and 0.044 mg/kg or 3.0 mg and 6.7 mg THC, IOP was reduced between 29% and 62%, respectively. The peak IOP reduction was described between 30 min and 90 min after administration [8]. In both documented studies, significant side effects, like euphoria, dizziness, confusion, and pre-syncopal symptoms, were reported [8,104,105].

### 5.2. Side Effects

The side effects of cannabinoids are related to the route of administration. Many of the reported studies in which cannabinoids were administered to lower IOP reported adverse effects in neurological, cardiovascular, ophthalmological, pulmonary, gastrointestinal, hepatic, renal, dermatological, or muscular systems after administration [34,106,107]. Although cannabinoids exert multiple effects on a variety of different organ systems, the acute psychological effects generally resolve within 2–6 h after application [10,106]. Neurologic side effects include dizziness, drowsiness, anxiety, euphoria, or hallucinations. Frequent digestive or cardiovascular reactions have been reported to include abdominal pain, nausea, vomiting, tachycardia, hyper- or hypotension, or syncope. As for ophthalmological side effects, conjunctival hyperemia, photosensitivity, and blurry vision may occur, thereby limiting long-term clinical use [34].

### 5.3. Novel Topical Formulations

Systemic side effects may be prevented or at least reduced by topical administration. However, to date, the topical application of cannabinoids continues to face limitations. Cannabinoids are highly lipophilic molecules with low aqueous solubility. After instillation, less than 5% of the applied dose reaches the intraocular tissues, as precorneal factors, such as drainage, non-corneal absorption, or induced lacrimation, limit absorption [56]. Studies which investigated the IOP-lowering effects of topical cannabinoids mostly failed to show significant effects [108,109,110,111]. Different galenical approaches to improve local availability include the use of sesame oil or mineral oil as vehicles for higher solubility, and newer drug delivery systems based on cyclodextrin, prodrugs, and nanoparticles for increased tissue penetration [56,112]. However, these tested formulations suffered from limited tolerability, such as burning sensation or lid swelling, which limited their long time use and enforced the development of better drug delivery vehicles [113,114].

Three studies that showed promising IOP-lowering or tissue-penetrating effects of cyclodextrins, prodrugs, and/or nanoparticles are presented below. In eight glaucoma patients, topical administration of WIN55212-2, a synthetic and selective CB_1_ receptor agonist, in combination with 2-hydroxylpropyl-β-cyclodextrin resulted in an IOP-lowering effect [91]. The authors described no major side effects and their solution demonstrated good stability and tolerability [8,91]. IOP was significantly reduced by 15 ± 0.5% (25 μg group) and 23 ± 0.9% (50 μg group) 30 min after administration [91]. The IOP-lowering effect was maximal 1 h after administration and tended to dissolve 2 h after administration [91]. The prodrug concept increases water solubility of Δ^9^-THC without undergoing great chemical modification. An investigated prodrug of Δ^9^-THC is Δ^9^-THC-valine-hemisuccinate (THC-VHS). Taskar et al. improved the effect of THC-VHS administered to normotensive rabbits using solid lipid nanoparticles (SLNs), which reportedly resulted in a reduction of IOP which was longer (480 min) than for 2.5% *w*/*v* pilocarpine hydrochloride (120 min) and 0.2% *w*/*v* timolol maleate (180 min) [115]. The tissue concentration of THC-VHS in the iris-ciliary bodies and retina–choroid was significantly higher with SLNs which may not only hold therapeutic potential for effects on IOP, but also neuroprotection [115]. Another nanoparticle formulation was used by Kabiri et al. [113]. The authors described a nanoparticle-laden hydrogel, where the hydrogel is composed of hyaluronic acid and methylcellulose, along with the amphiphilic nanoparticles of poly(ethylene oxide) and poly(lactic acid) [113]. They loaded the nanoparticles with cannabigerolic acid (CBGA), a close cannabinoid mimic molecule [113]. Corneal drug penetration was 300% higher in comparison to the control group [113]. The authors reported that only 0.015% of the applied CBGA load permeated through the cornea, which was justified through the absence of lachrymal drainage [113]. This method improved both bioavailability and corneal permeation, and reportedly reduced ocular irritation [113]. The amphiphilicity of these nanoparticles is well suited to transport lipophilic molecules, such as cannabinoids, through the cornea [113]. Recent developments of formulations improving the penetration of topically administered lipophilic molecules may be promising to evaluate the role of topically administered cannabinoids on IOP.

## 6. Neuroprotective Actions of Cannabinoids

Axonal injury can be mediated by ischemic effects, like peripapillary circulation abnormalities, or compression, e.g., through high IOP [116]. Neuroprotection is a strategy to prevent neuronal death, as neurons are postmitotic and cannot be replaced when lost [116]. Since glaucoma leads to a loss of RGCs and degeneration of the optic nerve head via apoptosis, much emphasis has been put into the development of neurodegenerative agents. To date, despite many efforts, no neuroprotective agent has been able to display clinically significant neuroprotective properties in a large, controlled trial or received drug approval from the regulatory agencies. This may be partly due to the fact that we do not yet fully understand why ganglion cells die in glaucoma and what would be an appropriate drug target for a potential neuroprotective approach. Among others, excitotoxicity, mitochondrial dysfunction, protein misfolding, oxidative stress, inflammation, and neurotrophin deprivation have been described as potential targets for neuroprotection in glaucoma [117,118].

### 6.1. Targeting Mechanisms in Neuroprotection

In the context of cannabinoids as potential neuroprotective agents, glutamate-induced excitotoxicity may be of particular interest. It has been well described that excessive stimulation of neurons by neurotransmitters, such as glutamate, can lead to neuronal death and is usually referred to as glutamate excitotoxicity [117,118]. Glutamate excess and N-methyl-D-aspartate (NMDA) receptor activation induces calcium influx and releases reactive oxygen species (ROS), leading to apoptosis [119]. Several studies have shown that cannabinoids prevent glutamate- or NMDA-induced neurotoxicity in isolated neurons or brains by activating CB_1_ receptors [120,121,122,123]. Thus, it has been hypothesized that activation of CB_1_ receptors may mediate neuroprotective effects in neurodegenerative diseases [124]. In contrast, CB_1_ receptors, which are not expressed in glutamatergic neurons, are thought to trigger cell death pathways and promote a pro-inflammatory response [125]. In glial cells, they may trigger reactive oxygen species/reactive nitrogen species (ROS/RNS) production and inflammatory cell activation [125]. Due to the diversity of these results, further studies are needed to evaluate the role of CB_1_ receptor signaling and ROS formation, which currently depends on the cell type and context [125].

In addition, the strong anti-inflammatory potential of the CB_2_ receptor signaling pathway makes cannabinoids another promising approach in neuroprotection [124]. Neuroinflammation is associated with microglial and/or astroglial cell activation triggered by pro-inflammatory factors, like cyto-/chemokines or elevated levels of ROS/RNS, ultimately leading to neuronal death [125]. The neuroprotective effect of CB_2_ receptor agonists in neurodegenerative diseases attenuates microglial activation by reducing ROS/RNS production, preventing leucocyte recruitment, attenuating vascular inflammation, and improving blood–brain barrier dysfunction [125]. Perivascular microglia exhibit high CB_2_ expression levels, suggesting that CB_2_ receptor activation improves the cerebral blood flow in neuroinflammatory diseases [126].

TRPV receptors are novel targets for developing treatment strategies in glaucoma. Since the TRPV1 receptor is located in neuronal tissues, such as the retina, this receptor has been mostly studied for its neuroprotective effects [60]. The death of RGCs is triggered at high doses of TRPV1 agonists, which can be explained by the following two mechanisms. Leonelli et al. suggested that this pro-apoptotic effect of TRPV1 under stress is dependent on nitric oxide synthase and NMDA-mediated glutamatergic excitotoxicity [127,128]. TRPV1 activation can be either be achieved through direct activation or indirectly through other endogenous ligands in the retina, such as endothelin-1 (ET-1) [129]. ET-1, which is upregulated in the glaucomatous eye, can potentiate the activity of TRPV1 [130]. In glaucoma patients, local expression of TRPV1 receptors in the retina is increased due to the high IOP [60,131]. TRPV1 receptors lead to an extracellular calcium influx, which in return leads to a net hyperpolarization on ganglion cell firing rates [60,131]. Overall, this protects the RGCs by reducing excessive neuronal cell activity and alleviating stress and metabolic demands on the cells [74,131]. Weitlauf et al. proved this hypothesis using mice that lacked TRPV1 receptors and showed that when increasing the IOP, TRPV1 -/- RGCs did not show any compensatory increase of TRPV receptors along with no according increase in firing rates [131]. This supports the idea that TRPV1 receptors promote neuronal survival in response to disease-relevant stressors through transient enhancement of local excitation at postsynaptic sites [131].

### 6.2. Evidence for Neuroprotection

As stated above, clinical evidence for the neuroprotective effects of cannabinoids in human studies is sparse. There are conceptual and methodological problems that make it difficult to transfer preclinical results to clinical glaucoma practice [132]. Reasons include the paucity of adequate animal models that can fully mimic human diseases along with the difficulty to predict the ocular bioavailability of drugs in humans [132,133]. Moreover, in human trials, the intervention is performed after diagnosis, whereas in most experimental studies, the neuroprotective agent is administered at the time of or prior to the injury [132]. Furthermore, outcome measures in the clinic are, for ethical reasons, functional outcomes that take weeks or months to show changes, whereas animal studies mostly employ histopathologic endpoints [132,133].

Preclinical studies have demonstrated neuroprotective effects of cannabinoids, but the specific mechanisms and therapeutic potential of the targeted receptors are not yet fully understood. Agonism and antagonism of the same receptors showed neuroprotective effects, which may depend on the used model, time of treatment, dosage, or individual differences [60]. Cannabinoid administration reduced RGC loss in rat animal models with episcleral vessel cauterization-induced glaucoma or saline-induced acute IOP elevation via CB_1_ receptor agonism [134,135,136,137]. Fourteen rats with unilateral glaucoma induced through cauterization of episcleral vessels received 5 mg/kg Δ^9^-THC or ethanol solution via intraperitoneal administration weekly for 20 weeks [134]. In this study, Δ^9^-THC induced a 10–20% reduction of RGC loss, while RGC loss was 40–50% in the control group [134]. Further animal models on rats examining optic nerve head neuroprotection were performed on optic nerve crush injury [138,139]. In an experimental rat model, reduction of injury-induced metabolic and electrophysiological deficits were achieved during a course of six hours after optic nerve axotomy [139]. A synthetic non-psychotropic cannabinoid and NMDA agonist, HU-211, was administered once intraperitoneally and yielded the positive result of regenerative growth and axonal sprouting 30 days after the initial injury, which in return was absent in control animals [138]. The neuroprotective effects of cannabinoids have been investigated in different animal models of ocular diseases and neurodegeneration [9,60]. In a streptozotocin-induced diabetic retinopathy rat model, CBD was found to reduce neurotoxicity, oxidative stress, damage to the blood–retinal barrier, inflammation, and retinal cell death through the inhibition of p38 MAP kinase [140]. In retinitis pigmentosa animal models, the synthetic cannabinoid HU-210 and a CB_1_ receptor antagonist SR141716A attenuated photoreceptor and retinal degeneration [141,142]. CB_1_ and CB_2_ receptor agonism via eCBs, 2-AG, or AEA hampers microglial activation and RGC loss in rat models, including the phosphoinositide 3-kinase (PI3K)/Akt signaling pathway [143,144]. In contrary, Maccarone et al. suggested the CB_1_, and more pronounced CB_2_ receptor antagonism as a possible neuroprotective mechanism since they reduced photoreceptor neurodegeneration [145]. CB_1_/CB_2_ receptor antagonism is similarly described and therefore it suggests a common pattern of response in retinal damage [145]. Since TRPV1 increases in RGCs due to elevated pressure in the eye, antagonism of the channel was able to reduce RGC apoptosis [146]. In contrast to antagonizing TRVP1, others have shown that TRPV1 receptor agonists can inhibit apoptosis in RGCs and protect against RGC inactivation [137,147]. Contrary to the studies mentioned, some models also reported neurodysfunctional effects due to cannabinoids and their receptors. Two months of systemic treatment with Δ^9^-THC resulted in toxic effects on photoreceptor cells in mice through functional loss in electroretinography and apoptosis [148]. For a detailed overview of other studies on neuroprotection in ocular diseases, the reader is referred to more extensive reviews [9,20,60]. An excellent overview on in vivo animal glaucoma models is provided by Gallo Afflitto et al. [60].

To date, the evidence from clinical trials for a potential neuroprotective effect of cannabinoids is sparse. PEA, an endocannabinoid-like molecule, showed promising effects in a single center, randomized, prospective, single-blinded, two treatment, two period crossover study by Rossi et al. [149]. The authors demonstrated that orally administered PEA doses of 600 mg/day for four months increased the electric activity of RGCs and retina that were measured using pattern evoked electroretinograms (PERGs) in patients with stable primary open-angle glaucoma and normal tension glaucoma. In addition, this study showed that PEA lowered the IOP significantly [149]. These authors discussed that both IOP-lowering and direct neuroprotective effects are involved in RGC survival, and in this way, influence each other [149]. It has previously been shown that PEA reduces IOP in glaucoma patients and that this indirectly contributes to neuroprotection [149]. Alternatively, PEA showed vasoactive properties through vasorelaxation of the ophthalmic artery via PPARα transcription factors in other studies, which suggests that the neuroprotective effect of cannabinoids may be related to their vasodilatation capabilities [8,150].

Challenging the hypothesis of a neuroprotective effect of cannabinoids, there is also evidence indicating that cannabinoids might be harmful and may lead to neuroretinal dysfunction. Studies by Schwitzer et al. showed delayed responses in ganglion cells and bipolar cells in electroretinography and delayed transmission of visual information in regular cannabis users [151,152,153]. Increased retinal background noise, as resting-state neural activity, was increased in regular cannabis users [154]. In most studies, the dosage of the cannabinoid was difficult to compare as it was self-reported by the subjects [155]. However, these studies might be limited by the concomitant use of other drugs, such as alcohol and tobacco, which may thereby limit the general applicability of the results [155]. As mentioned for the preclinical studies, the conflicting results of these clinical studies also suggest that the molecular mechanisms and effects of cannabinoids on ocular structures and visual function are poorly understood, and further trials are required.

## 7. Vascular Targets of Cannabinoids and the Effect on Ocular Hemodynamics

**Scheme 1:**
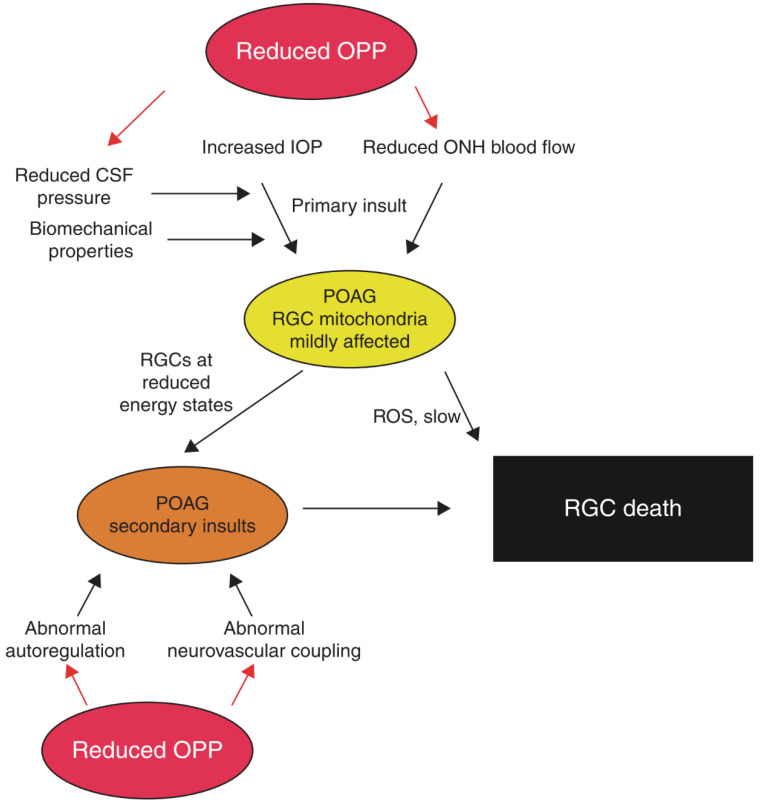
This overview shows the primary and secondary insults in glaucoma pathogenesis influenced by reduced ocular perfusion pressure (OPP). Elevated intraocular pressure (IOP) and optic nerve head (ONH) ischemia lead to the primary insult at the optic nerve head. Biomechanical properties of ocular tissues and reduced cerebrospinal fluid (CSF) can alter susceptibility. In patients with primary open-angle glaucoma (POAG), retinal ganglion cells (RGCs) then exhibit reduced energy states as the mitochondria are affected. The combination of oxidative stress caused by reactive oxygen species (ROS) and secondary insults, like abnormal autoregulation and abnormal neurovascular coupling, may lead to RGC death, which is the end of the pathway. “Reprinted with permission form Ref. [14]. 2013, Elsevier”.

In addition to IOP, it has been hypothesized that vascular risk factors play a role in the pathogenesis in glaucoma [156]. The hypothesis that vascular impairment may contribute to the pathogenesis of glaucomatous optic neuropathy implies that ganglion cell damage is a consequence of vascular dysregulation and/or insufficient blood supply in the optic nerve head [14,157,158,159,160]. Using a variety of different methods, it has been shown that ocular perfusion and oxygen extraction are reduced in patients with glaucoma, indicating that local hypoxia may play a role in the pathogenesis of this disease [161,162,163,164,165]. However, it has also been suggested that glaucoma is associated with dysregulated perfusion rather than reduced blood flow [159,166]. As such, there is evidence that vascular autoregulation, an intrinsic mechanism of the retinal vasculature to keep the perfusion pressure constant, may be compromised in patients with glaucoma [167]. Along this line of thought, previous evidence indicated an abnormal correlation between optic nerve head perfusion and ocular perfusion pressure in patients with glaucoma [168], which was normalized when IOP was lowered [169]. Further, neuro-vascular coupling is reduced in patients with glaucoma, an effect that seems to be largely independent from IOP [170]. For an in-depth discussion regarding the role of ocular perfusion in glaucoma, the reader is referred to previously published articles (as outlined in Scheme 1) [14,159,171].

### 7.1. Vascular Targets

The effects of cannabinoids on the ocular vasculature are insufficiently described. A full overview of the vascular targets of cannabinoids is beyond the scope of this review and the reader is referred to comprehensive recent review articles [172,173,174]. In the following, a short overview of the most important vascular drug targets and mechanisms has been provided.

Vascular responses seem to be mainly mediated through the involvement of the cannabinoid receptors CB_1_ and CB_2_, as well as non-cannabinoid receptors, such as the endothelial cannabinoid receptor (CB_e_) and vanilloid receptors [172,174,175]. In addition, cannabinoids influence the actions of vasoactive compounds, like angiotensin II, acetylcholine, methoxamine, and thromboxane receptor agonists [172]. Cannabinoids induce the lowering of blood pressure and vasorelaxation [175]. The proposed mechanisms have been mainly studied with regard to CB_1_ receptors in resistance vessels [173]. Most studies have shown that cannabinoids mediate vasorelaxant effects at least partially via CB_1_ receptors [172]. The definitive pathway is not completely understood but depending on receptor location in the perivascular nerves or in the blood vessels, two possible mechanisms are suggested. When the CB_1_ receptors are located in the perivascular nerves, they inhibit the release of norepinephrine from sympathetic nerve terminals [175]. Depending on the location of the CB_1_ receptors in the smooth muscle cell layer or in the endothelial cells, different intracellular pathways will be activated. Pathways that activate the CB_1_ receptor in the smooth muscle cells include the inhibition of L-type calcium channels, adenylyl cyclase, and initiation of MAPK-mediated cascades. Activation of the CB_1_ receptor in endothelial cells induce the synthesis or release of nitric oxide, endothelial-derived hyperpolarizing factors, or prostacyclin [173].

CB_2_ receptors also promote vasorelaxation, but not much is currently known about the molecular pathways. Here, it has been hypothesized that activation of phospholipase C and formation of inositol 1,4,5-triphosphate mediates vasorelaxation [176].

As previously mentioned, CB_1_ and CB_2_ receptors inhibit adenylyl cyclase. In contrast, noradrenaline, as part of the sympathetic nervous system, activates adenylyl cyclase, which results in the release of calcium and vasoconstriction. These two counterregulatory systems indicate that cannabinoids through the activation of one system lead to the inhibition of the second counterregulatory system [177]. With simultaneous upregulation of the ECS, the sympathetic nervous system and vasoconstrictors of the renin–angiotensin–aldosterone system (RAAS) appear to be oppositely regulated [177].

Furthermore, eCBs regulate vasoactivity not only in the peripheral vasculature, but also in the CNS microvasculature [69]. Previous studies described that the effects of the eCB AEA on vessels are not only attributed to the CB_1_ receptor [178]. As the vascular tissue tone depends on ET-1 and NO, an in vitro study by Ronco et al. examined the effects of the endogenous cannabinoid receptor agonist AEA in human endothelial cells. The authors speculated that AEA leads to vasodilatation via non-CB_1_ receptor-dependent inhibition of ET-1 production and CB_1_-mediated increase of NO, in this way, increasing the vasodilatation/vasoconstriction ratio [179]. In another in vitro study by MacIntyre et al., ET-1-mediated vasoconstriction in retinal arterioles was shown to be inhibited by abnormal CBD (Abn-CBD), a CBD analog and agonist at the putative O-1918-sensitive endothelial cannabinoid receptor (CB_e_). Virtually similar effects have been demonstrated for N-arachidonoyl glycine, an endogenous eCB and putative agonist for GPR18, which the authors detected in the endothelium of retinal vessels [69]. This indicates that cannabinoids mediate changes in vascular resistance through endothelium-dependent signaling mechanisms on a local basis independent of nitric oxide and involving novel endothelial non-CB_1_/CB_2_ receptor targets [180]. These findings may be of particular clinical interest since ET-1 is a highly potent vasoconstrictor and its levels are elevated in glaucoma patients and animal models of glaucoma [22]. The endothelin receptors 1A and 1B are both present in the region of the optic nerve head [181].

### 7.2. Evidence for Hemodynamic Effects

When cannabinoids and eCBs are applied to isolated arteries or perfused vascular beds, they exhibit changes in vascular resistance [172,174]. In vitro studies with eCBs and synthetic cannabinoid agonists in isolated arteries showed predominantly vasorelaxant effects, but there is also evidence for vasoconstrictor responses [172,174]. In the bovine ophthalmic artery, the endogenous cannabinoid ligand AEA and the synthetic cannabinoid agonist WIN55212-2 induced relaxation through involving the CB_1_ receptor-sensitive pathway [182]. Furthermore, it has been described that the effect on the vessels depends on the basal vascular tone, since in isolated retinal arterioles vasodilatory actions were only shown in precontracted vessels [180].

In anesthetized animals, cannabinoids induced prolonged hypotension, whereas pressor responses were noted in conscious animals [174]. The reason for these differing results may be related to the fact that the effects of cannabinoids appear to be dependent on the species, type of cannabinoids, and experimental preparation [172,183]. In a study with rabbits, Δ^9^-THC increased blood flow in the iris, ciliary processes, and choroid, but not the retina [184]. This was interpreted by the authors as dilatation of vessels leading away from the anterior uvea. Administration of Δ^9^-THC leads to vasodilatation of conjunctival vessels and redness of the eye, demonstrating that cannabinoids exert vasoactive effects on the anterior eye segment [185].

Much evidence of blood flow alteration using cannabinoids has been studied in the cerebral circulation. As the optic nerve is part of the CNS, these findings may be of interest for blood flow in the retina or optic nerve head. A large systematic review by Ogunbiyi et al. summarized the effects of inhaled and intravenous THC on cerebral blood flow in human and animal studies [186]. Notably, acute THC administration caused an increase in the cerebral blood flow, whereas chronic cannabis use resulted in a decrease in the cerebral blood flow [186]. The authors attributed a vasodilatory effect for the increase in blood flow and a downregulation of CB_1_ receptors as tolerance after prolonged exposed activation, resulting in an opposite effect. This effect of decreased cerebral blood flow in chronic cannabis users can be reversed after prolonged abstinence from the drug [186].

**Figure 2:**
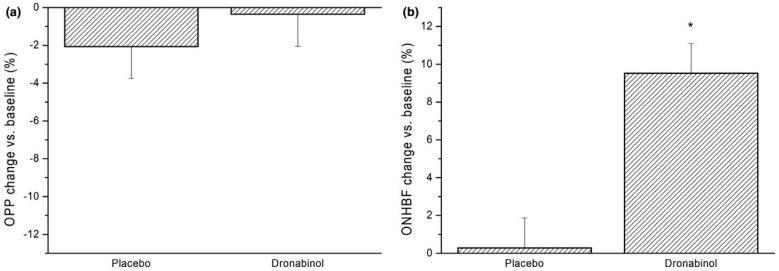
Dronabinol and optic nerve head blood flow in healthy subjects. On the left side (**a**), the relative changes in OPP after administration of placebo and dronabinol (−2.1 ± 8.4% vs. −0.4 ± 8.2%, respectively, *p* = 0.48 between study days) are shown. On the right side (**b**), the relative change in optic nerve head blood flow (ONHBF) at rest was increased by 9.5 ± 8.1% after dronabinol intake (*p* < 0.001). Data are presented as mean ± SD (*n* = 24). * Significant changes vs. baseline. “Reprinted from Ref. [188]. This work is licensed under the Creative Commons Attribution 4.0 International (CC BY 4.0) license. To view a copy of this license, visit http://creativecommons.org/licenses/by/4.0/”.

With respect to retinal hemodynamics, it has been reported that orally administered 7.5 mg dronabinol resulted in a significant decreased retinal arteriovenous passage time from 1.77 ± 0.35 s to 1.57 ± 0.31 s (*p* = 0.028) in eight healthy subjects without adverse respiratory or cardiovascular side effects [96], indicating an increase in retinal blood flow. In another study, the arteriolar and venular diameters of 55 frequent cannabis users and 51 control subjects were compared using retinal imaging [187]. Cannabis users had, on average, larger arteriolar diameters, which was interpreted as a retinal vasodilatory effect of cannabinoids [187]. A study performed in our laboratory revealed that the oral intake of 5 mg dronabinol significantly increased optic nerve head blood flow in 24 healthy subjects without affecting the retinal autoregulatory response to increased perfusion pressure (Figure 2) [188]. Whether cannabinoids may be a future option to therapeutically increase ocular perfusion in patents with glaucoma or other ocular vascular disorders has yet to be clarified [188].

## 8. Conclusions and Future Perspectives

The potential of cannabinoids to lower IOP is limited due to their short-lasting effects and systemic toxicity, as high doses are often required. Overall, there is weak evidence that cannabinoids other than THC, which is mainly responsible for psychotropic side effects, can lower intraocular pressure. For this reason, cannabinoids are not currently recommended for clinical use in glaucoma. To counteract the psychotropic effects, further research is required on non-psychotropic cannabinoids in neurodegenerative diseases. Moreover, new promising molecules for topical application of cannabinoids are in their late stages of testing. In addition, neuroprotective effects of cannabinoids appear promising in animal models, but conflicting results indicate that the mechanisms on neuronal survival are poorly understood. Nevertheless, the anti-oxidative, anti-inflammatory, and vasodilatory properties may represent a new opportunity for neurodegenerative diseases, including glaucoma. Further studies under clinical conditions with larger populations and longer follow-up times would be needed to find out more about the molecular mechanisms and long-term effects of cannabinoids in glaucoma patients.

## Data Availability

Not applicable.
